# Antimicrobial resistance in commensal *Escherichia coli* and *Enterococcus* spp. is influenced by production system, antimicrobial use, and biosecurity measures on Spanish pig farms

**DOI:** 10.1186/s40813-021-00206-1

**Published:** 2021-03-19

**Authors:** Oscar Mencía-Ares, Héctor Argüello, Héctor Puente, Manuel Gómez-García, Edgar G. Manzanilla, Avelino Álvarez-Ordóñez, Ana Carvajal, Pedro Rubio

**Affiliations:** 1 Department of Animal Health, Faculty of Veterinary, Universidad de León, León, Spain; 2 Animal and Grassland Research and Innovation Centre, Teagasc, Moorepark, Fermoy, Ireland; 3 School of Veterinary Medicine, University College Dublin, Dublin, Ireland; 4 Department of Food Hygiene and Technology, Faculty of Veterinary, Universidad de León, León, Spain; 5 Institute of Food Science and Technology, Universidad de León, León, Spain

**Keywords:** Antibiotic usage, Bioindicator, *Enterococcus* spp., *Escherichia coli*, One health, Swine, Sustainable farming

## Abstract

**Background:**

Antimicrobial resistance (AMR) is a global public health threat consequence of antimicrobial use (AMU) in human and animal medicine. In food-producing animals factors such as management, husbandry or biosecurity may impact AMU. Organic and extensive Iberian swine productions are based on a more sustainable and eco-friendly management system, providing an excellent opportunity to evaluate how sustained differences in AMU impact the AMR in indicator bacteria. Here, we evaluate the usefulness of commensal *Escherichia coli* and *Enterococcus* spp. isolates as AMR bioindicators when comparing 37 Spanish pig farms from both intensive and organic-extensive production systems, considering the effect of AMU and biosecurity measures, the last only on intensive farms.

**Results:**

The production system was the main factor contributing to explain the AMR differences in *E. coli* and *Enterococcus* spp. In both bacteria, the pansusceptible phenotype was more common (*p* < 0.001) on organic-extensive farms when compared to intensive herds. The microbiological resistance in commensal *E. coli* was, for most of the antimicrobials evaluated, significantly higher (*p* < 0.05) on intensive farms. In enterococci, the lincosamides usage revealed the association between AMR and AMU, with an increase in the AMR for erythromycin (*p* < 0.01), quinupristin-dalfopristin (*p <* 0.01) and the multidrug-resistant (MDR) phenotype (*p* < 0.05). The biosecurity measures implemented on intensive farms influenced the AMR of these bioindicators, with a slightly lower resistance to sulfamethoxazole (*p* < 0.01) and the MDR phenotype (*p* < 0.05) in *E. coli* isolated from farms with better cleaning and disinfection protocols. On these intensive farms, we also observed that larger herds had a higher biosecurity when compared to smaller farms (*p* < 0.01), with no significant associations between AMU and the biosecurity scores.

**Conclusions:**

Overall, this study evidences that the production system and, to a lesser extent, the biosecurity measures, contribute to the AMR development in commensal *E. coli* and *Enterococcus* spp., with antimicrobial usage as the main differential factor, and demonstrates the potential value of these bacteria as bioindicators on pig farms in AMR surveillance programs.

## Background

Antimicrobial resistance (AMR) is one of the largest threats to global health and food security [1]. The most important single factor that leads to AMR is antimicrobial use (AMU) [2] and, although its use in human medicine is the main driver of AMR, AMU in veterinary medicine also contributes to the burden of AMR in human health [3]. The long-term AMU in food-producing animals facilitates the development and spread of AMR bacteria through food, water or slurry, which is used as fertilizer. In fact, similarities among AMR bacteria in humans and animals have been observed in foodborne pathogens and commensal bacteria, such as *E. coli*, *Enterococcus* spp. or *Salmonella* spp. [4].

Despite antimicrobials are essential in bacterial diseases treatments [5], current policies aim at reducing AMU in livestock [6], particularly in the swine industry, which is the most extensive agricultural user of antimicrobials in the European Union [7, 8]. In this sense, the AMR surveillance in targeted zoonotic or bioindicator bacteria through European programs [9] constitutes a fundamental pillar in the evaluation of the trends in AMR due to antimicrobial selection pressure [10].

Reductions in AMU may be achieved through the improvement of vaccination programs and biosecurity standards [11, 12], and strategies which involve animal husbandry and welfare, such as alternative farming systems as it has been observed on organic pig farms [13]. In this sense, within the Spanish swine production, the extensive system is a traditional and sustainable production system associated with the Iberian pig which uses natural resources and integrates pig production into an oak field ecosystem [14]. Thus, this management system provides an excellent opportunity to compare AMR patterns in potential bioindicator bacteria in pigs and farm environments compared to those of intensive production farms. Hence, this study aims at determining the fitting of *Escherichia coli* and *Enterococcus* spp. isolates as AMR bioindicator species in a comparative study between intensive and organic-extensive Spanish swine herds, considering AMU and biosecurity score, the last only on intensive pig farms.

## Methods

### Farm selection and sample collection

A total of 37 swine farms were selected from different Spanish regions and according to their productions system to represent a convenience sample of Spanish intensive (18 herds), extensive (12 herds) and organic (7 herds) management systems. Organic and extensive farms were merged into a single category as organic herds were mainly converted from extensive farms, rearing Iberian pig on a system based on the use of natural resources in farrow-to-finish farms. Thus, the farms were grouped into intensive (18 herds) and organic-extensive (19 herds) for further analyses.

Sampling and farm characteristics are detailed in Mencía-Ares et al. [15]. Briefly, sampling was carried out from 2017 to 2018 in pigs in the last month of the fattening period, with no antimicrobial treatment in the immediate month prior to the sampling. On each fattening unit, faeces, environmental swabs and slurry, when available, were collected.

### Antimicrobial use

The veterinary practitioner responsible for each farm recorded AMU on the pigs in the sampled fattening unit during the immediate four-month period prior to sampling. This record was based on the register of treatments. Antimicrobial use was categorized into 13 classes: (i) total, (ii) penicillins, (iii), third generation cephalosporins, (iv) aminoglycosides, (v) macrolides, (vi) lincosamides, (vii) quinolones, (viii) tetracyclines, (ix) phenicols, (x) polymyxins, (xi) sulfonamides, (xii) diaminopyrimidines, and (xiii) pleuromutilins. For each antimicrobial class, usage per farm was expressed in annual mg/PCU, following the European Surveillance of Veterinary Antimicrobial Consumption (ESVAC) protocol [16]. Total AMU per farm was calculated as the sum of the individual contributions of each antimicrobial class on each farm.

### Bacterial isolation and characterization

*Escherichia coli* isolation was performed with a direct inoculation of the samples with a swab in MacConkey agar (Scharlau, Sentmenat, Spain) at 37 °C for 24 h. Presumptive colonies were subcultured in tryptic soy agar (Scharlau, Sentmenat, Spain) at 37 °C for 24 h.

Samples were directly inoculated with a swab in Slanetz and Bartley agar (Oxoid, Basingstoke, UK) at 37 °C for 48 h for *Enterococcus* spp., isolation. Dark pink colonies were subcultured in bile esculin azide agar (Merck, Darmstadt, Germany) at 44 °C for 4 h for *Enterococcus* confirmation. Presumptive colonies were subcultured in brain heart infusion (BHI) agar (Merck, Darmstadt, Germany) at 37 °C for 24 h.

*E. coli* and *Enterococcus* presumptive isolates were confirmed and characterized at species level, respectively, with MALDI-TOF mass spectrometry using the IVD MALDI Biotyper (Bruker Daltonik, Bremen, Germany) and following the manufacturer’s standard protocols.

### Antimicrobial susceptibility testing

Antimicrobial susceptibility testing was conducted on a single isolate from each type of sample (faeces, slurry and environment) and an extra isolate randomly selected from any of the three sample types. The technique followed the procedures outlined by the European Committee on Antimicrobial Susceptibility Testing (EUCAST) [17]. The minimum inhibitory concentration (MIC) of the tested antimicrobials was determined using the broth microdilution method. The “microbiological” resistance was determined in accordance with the epidemiological cut-off value (ECOFF), thus dividing the microorganisms depending on whether they have (non-wild type, NWT) or not (wild type, WT) acquired resistance mechanisms to each antimicrobial [18]. Non-wild type and resistant phenotype will be indistinctly used throughout the study. Multidrug-resistance (MDR) was defined as acquired non-susceptibility to at least one agent in three or more antimicrobial classes [19]. A microorganism susceptible to all antimicrobials tested was defined as pansusceptible (PNS).

AMR in *E. coli* and *Enterococcus* spp. isolates was evaluated with EUVSEC and EUVENC Sensititre plates (TREK Diagnostic Systems, East Grinstead, UK), respectively. The antimicrobials evaluated and their ECOFFs are shown in Tables 1 and 2. *Escherichia coli* ATCC 25922 and *Enterococcus faecalis* ATCC 29212 were used as control strains, respectively.

**Table 1:**
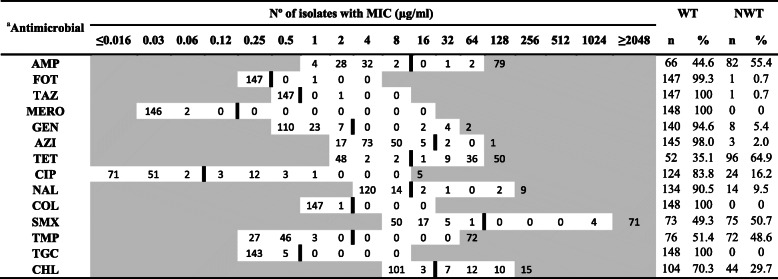
Minimum inhibitory concentrations (MICs) of 14 antimicrobials against 148 *Escherichia coli* isolates from animal and environmental samples recovered from swine farms. The thick line represents the epidemiological cut-off value (ECOFF) used for each antimicrobial to classify isolates into non-wild type (NWT) and wild type (WT). Areas in grey represent values outside the concentrations included in the broth microdilution method

^a^Antimicrobial: *AMP* Ampicillin, *FOT* Cefotaxime, *TAZ* Ceftazidime, *MERO* Meropenem, *GEN* Gentamicin, *AZI* Azithromycin, *TET* Tetracycline, *CIP* Ciprofloxacin, *NAL* Nalidixic acid, *COL* Colistin, *SMX* Sulfamethoxazole, *TMP* Trimethoprim, *TGC* Tigecycline, *CHL* Chloramphenicol

**Table 2:**
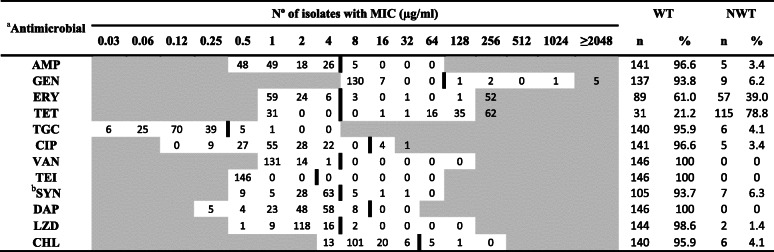
Minimum inhibitory concentrations (MICs) of 12 antimicrobials against 146 *Enterococcus* spp. isolates from animal and environmental samples recovered from swine farms. The thick line represents the epidemiological cut-off value (ECOFF) used for each antimicrobial to classify isolates into non-wild type (NWT) and wild type (WT). Areas in grey represent values outside the concentrations included in the broth microdilution method

^a^Antimicrobial: *AMP* Ampicillin, *GEN* Gentamicin, *ERY* Erythromycin, *TET* Tetracycline, *TGC* Tigecylince, *CIP* Ciprofloxacin, *VAN* Vancomicin, *TEI* Teicoplanin, *SYN* Quinupristin-dalfopristin, *DAP* Daptomycin, *LZD* Linezolid, *CHL* Chloramphenicol

^b^SYN: *Enterococcus faecalis* was excluded of quinupristin-dalfopristin MIC distribution, WT and NWT phenotypes due to its intrinsic resistance

Prior to testing, *E. coli* and *Enterococcus* spp. colonies cultured in BHI agar at 37 °C for 24 h were suspended in 5 ml of demineralized water to reach a turbidity of McFarland 0.5. Ten microliters of the bacterial suspension were transferred to 11 ml of Mueller-Hinton broth (TREK Diagnostic Systems, East Grinstead, UK) and 50 μl per well were dispensed with the Sensititre AIM Automated Inoculation Delivery System (TREK Diagnostic Systems, East Grinstead, UK). Plates were sealed and incubated in aerophilic atmosphere at 37 °C for 24 h.

### Biosecurity assessment

To evaluate the biosecurity in the intensive herds, a pre-established protocol, the Biocheck.UGent™ scoring system, developed by Gent University [20], was used. A detailed description of this questionnaire can be accessed elsewhere [21–23]. Briefly, it contains 109 closed questions grouped into internal and external biosecurity, with six subcategories in each group. Each subcategory includes different practices and its score is given in a rank from 0 (worst scenario) to 100 (best scenario). Each question has a fixed score. Internal and external biosecurity scores are determined by the weighted average of the subcategories, as it was established by Laanen et al. [22]. Total biosecurity score is the result of the average of internal and external biosecurity scores.

The questionnaire was translated from English into Spanish and the questions were answered using an online platform [24] by the manager of the pig farm. Results were transcribed to the Biocheck.Ugent™ online database and an Excel sheet (Microsoft Office). The final scores for each biosecurity category were obtained for each farm and were used for further analyses.

The particular characteristics of the organic-extensive herds, their management and their husbandry practices did not conform to most of the Biocheck.Ugent™ sections and we failed to include the biosecurity scores of these herds. For this reason, biosecurity analyses were restricted to intensive herds.

### Statistical analysis

#### Mixed-effects logistic regressions

A database including farm characteristics, AMU (i.e. total values and specific consumptions for each antimicrobial class), biosecurity scores and AMR phenotypic data was created in an Excel sheet (Microsoft Office). The database was introduced into R version 3.6.2 [25] where all statistical analyses were conducted.

As a first step, descriptive analyses were performed to identify low variability in AMR. Thus, for those antimicrobials that had less than 5% of isolates classified as WT or NWT no further analyses were carried out due to lack of variability [26]. Quantitative variables were transformed to a log_10_ scale. Production system (intensive/organic-extensive), sample type (faeces/slurry/environmental), total AMU and AMU divided by antimicrobial classes were included as independent variables in a mixed-effects logistic regression to evaluate their influence in the occurrence of NWT, MDR and PNS phenotypes for the antimicrobials evaluated. Farm was introduced in the model as a random effect. For intensive farms, biosecurity scores were included as independent variables. Quinupristin-dalfopristin was excluded for the characterization of MDR and PNS phenotypes in *E. faecalis* due to its intrinsic resistance [27].

In each model, all variables were initially tested using an univariate mixed-effects logistic regression using the package *lme4* [28]. Predictor variables with *p* ≤ 0.10 in the likelihood ratio test (LRT) were considered for inclusion in subsequent multivariate analysis. In the multivariate analysis, all the combinations of fixed effects selected from the univariate analyses were run and ranked by the Akaike information criterion (AIC) using *dredge* function from *MuMIn* package [29]. The statistical models with ΔAIC ≤2 were individually analyzed and a model with *p* ≤ 0.05 in the LRT for each independent variable with a variance inflation factor ≤ 3.3 was selected as final model. Results for the fixed effects were reported as odds ratio (OR) including its 95% confidence interval (95% CI).

#### Clustering of AMR patterns

A clustering of AMR patterns was performed for *E. coli* and *Enterococcus* spp. isolates. Taking into account the chemical structure of the antimicrobials tested, an isolate was considered microbiologically resistant to an antimicrobial class if it was resistant to at least one member of such class. For both bacterial genera, isolates were clustered according to their AMR pattern using the unweighted pair group method with arithmetic mean (UPGMA) as hierarchical clustering method. *Enterococcus* isolates were divided in *E. faecalis* and enterococci other than *E. faecalis,* excluding the streptogramins antimicrobial class in *E. faecalis* clustering due to its intrinsic resistance to quinupristin-dalfopristin. The *pheatmap* package [30] was used for the representation of the clustered heatmaps of isolates. The AMR comparison between isolates from the two main clusters was carried out with the Chi-Square test.

#### Association between AMU and biosecurity

The association between AMU and biosecurity scores on intensive swine farms was initially tested with the pairwise Spearman’s rank correlation. Correlations were removed if *p* > 0.05, adjusting this *p-*value to avoid false positives using the Benjamini & Hochberg method [31]. The correlations were carried out with the *Hmisc* package [32]. Biosecurity scores were afterwards included as independent variables in a generalized gamma regression to evaluate their influence in the total AMU following the methods described in the mixed-effects logistic regression.

#### Principal component analysis

For the evaluation of the biosecurity on intensive swine farms, a principal component analysis (PCA) was performed on the biosecurity subcategories and the two main dimensions for the principal components were characterized. Farms were clustered by their biosecurity practices using the UPGMA as hierarchical clustering method. Biosecurity scores, number of fattening pigs per feedlot, total AMU and AMU divided by antimicrobial classes for each cluster were compared by Wilcoxon signed rank test.

## Results

### *Escherichia coli* antimicrobial resistance

**Fig. 1:**
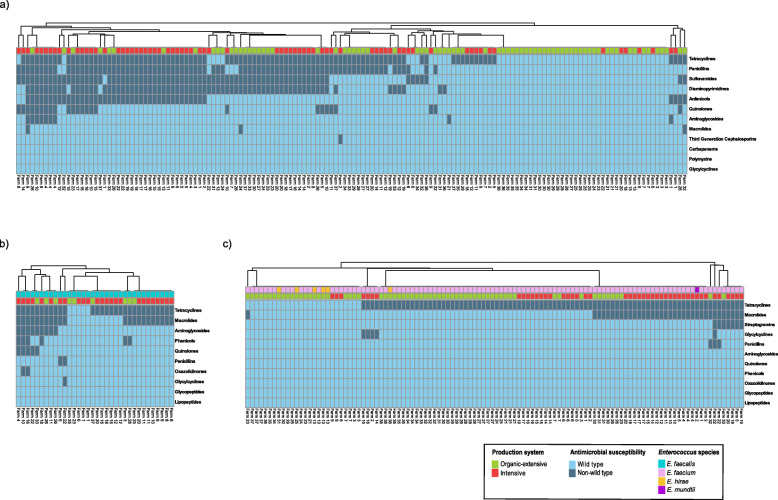
Antimicrobial resistance pattern in **a** *Escherichia coli* (148), **b** *Enterococcus faecalis* (34) and **c** enterococci other than *E. faecalis* (112) recovered from 37 Spanish pig farms. The isolates were clustered according to their antimicrobial resistance pattern using the unweighted pair group method with arithmetic mean (UPGMA) as hierarchical clustering method. For this purpose, the *pheatmap* package (Kolde, 2019) was used. Streptogramins antimicrobial class was excluded from the *E. faecalis* clustering due to its intrinsic resistance

A total of 148 isolates were recovered from faeces, slurry and environmental samples from 37 farms, with four isolates per herd. The MICs for these *E. coli* isolates are shown in Table 1. While 108 isolates (73.0%) were resistant to at least one antimicrobial, with 78 isolates (52.7%) defined as MDR, 40 isolates (27.0%) were characterized as PNS. There were 25 MDR patterns, being the most common the combination of penicillins, tetracyclines, sulfonamides, diaminopyrimidines and phenicols (28.2%), followed by the combination of penicillins, tetracyclines, sulfonamides and diaminopyrimidines (20.5%). NWT phenotype was mainly detected for tetracycline (64.9%) and ampicillin (55.4%), followed by sulfamethoxazole (50.7%) and trimethoprim (48.6%). Less frequent were the resistances to chloramphenicol (29.7%), ciprofloxacin (16.2%) or nalidixic acid (9.5%). Only one *E. coli* isolate exhibited AMR to extended-spectrum beta-lactamases by its lack of susceptibility to ampicillin, cefotaxime and ceftazidime. All isolates were susceptible to meropenem, tigecycline or colistin. As shown in Fig. 1a, AMR clustering of *E. coli* categorizes the isolates into two main clusters, mainly determined by the resistance to sulfonamides and diaminopyrimidines (*p* < 0.001). Most of the isolates resistant to sulfonamides, diaminopyrimidines, quinolones and phenicols were recovered from intensive farms.

When investigating the factors associated with the AMR to the antimicrobials evaluated, as MDR and PNS phenotypes, the only factor identified was the production system. NWT phenotype was less frequent in isolates from organic-extensive farms for ampicillin (*p* < 0.001; OR = 0.15; 95% CI = 0.05–0.36), tetracycline (*p* < 0.001; OR = 0.06; 95% CI = 0.01–0.19), ciprofloxacin (*p* < 0.01; OR = 0.07; 95% CI < 0.01–0.38), nalidixic acid (*p* < 0.05; OR = 0.05; 95% CI < 0.01–0.67), sulfamethoxazole (*p* < 0.001; OR = 0.16; 95% CI = 0.05–0.40), trimethoprim (*p* < 0.001; OR = 0.18; 95% CI = 0.06–0.43) and chloramphenicol (*p* < 0.001; OR = 0.01; 95% CI = 0.01–0.19), as it was the MDR (*p* < 0.001; OR = 0.13; 95% CI = 0.04–0.33) phenotype. PNS isolates were also more frequent in these organic-extensive herds (*p* < 0.001; OR = 5.61; 95% CI = 2.91–12.17). No associations were observed for the antibiotic gentamicin.

In the 72 *E. coli* isolated on intensive farms, the factors associated with the AMR revealed that ciprofloxacin resistance was associated with high phenicols consumption (*p* < 0.05; OR = 6.30; 95% CI: 1.04–80.40), while chloramphenicol resistance was linked to a high quinolone consumption (*p* < 0.05; OR = 5.38; 95% CI: 1.02–46.13). An increase in the biosecurity score for feed, water and equipment supply was slightly associated with a lower gentamicin resistance (*p* < 0.05; OR = 0.91; 95% CI: 0.78–0.98), as it was observed for cleaning and disinfection with sulfamethoxazole resistance (*p* < 0.01; OR = 0.96; 95% CI: 0.92–0.99) and the MDR pattern (*p* < 0.05; OR = 0.97; 95% CI: 0.93–0.99). Tetracycline resistance was slightly associated with a better disease management (*p* < 0.05; OR = 1.04; 95% CI: 1.00–1.11). No associations were observed for ampicillin, trimethoprim or nalidixic acid, as the PNS phenotype.

### *Enterococcus* spp. antimicrobial resistance

A total of 146 *Enterococcus* spp. isolates from faeces, slurry and environmental samples were further typed and tested for antimicrobial resistance. In these isolates, the predominant species were *E. faecium* (105) and *E. faecalis* (34), being less frequent *E. hirae* (6) and *E. mundtii* (1).

The MICs of these isolates are shown in Table 2. Twenty-seven isolates (18.5%) were defined as PNS, while 119 (81.5%) exhibited resistance to at least one antimicrobial, with 21 isolates (14.4%) characterized as MDR. The tetracyclines, macrolides and streptogramins MDR combination was the most frequent (23.8%) among the 11 MDR patterns observed. The NWT phenotype was predominant for tetracycline (78.8%), followed at a distance by erythromycin (39.0%). Less frequent was any observation of resistance to gentamicin (6.2%), tigecycline (4.1%), chloramphenicol (4.1%), ciprofloxacin (3.4%), ampicillin (3.4%) and linezolid (1.4%). In enterococci other than *E. faecalis*, quinupristin-dalfopristin resistance was uncommon (6.3%). All isolates were susceptible to daptomycin, vancomycin and teicoplanin. In *E. faecalis* and enterococci other than *E. faecalis* two main clusters were appreciated when grouping isolates by their AMR pattern whether they had or not a MDR phenotype (Fig. 1b and Fig. 1c).

The factors significantly associated with AMR to tetracycline, erythromycin and quinupristin-dalfopristin, as MDR and PNS patterns are shown in Table 3. PNS phenotype was more frequent on organic-extensive farms (*p* < 0.001). Tetracycline NWT phenotype was less common in organic-extensive herds (*p* < 0.001). Erythromycin resistance was more frequent in *Enterococcus* recovered from slurry samples (*p* < 0.05) and it was associated with a higher lincosamides (*p* < 0.01), penicillins (*p* < 0.01) and phenicols (*p* < 0.01) usage. The MDR phenotype was also more common in herds with high lincosamides (*p* < 0.001) and phenicols (*p* < 0.01) use. Quinupristin-dalfopristin NWT phenotype in enterococci other than *E. faecalis* was linked to farms with high lincosamides use (*p* < 0.001). No associations were observed for the antibiotic gentamicin.

**Table 3:** Factors associated with the non-wild type (NWT) phenotype for erythromycin, tetracycline, quinupristin-dalfopristin, multidrug-resistance and pansusceptible profile in 146 *Enterococcus* isolates recovered from 37 Spanish pig farms

		^a^Antimicrobial				
**Independent variable**		**ERY**	**TET**	^**c**^**SYN**	^**d**^**MDR**	^**d**^**PNS**
		**OR (95% CI)**	**OR (95% CI)**	**OR (95% CI)**	**OR (95% CI)**	**OR (95% CI)**
**Management system**	Intensive (Ref. category)	^b^NI		^b^NI	^b^NI	
	Organic-extensive		0.11 (0.02–0.35)			9.47 (2.66–59.26)
**Type of sample**	Faeces (Ref. category)		^b^NI	^b^NI	^b^NI	^b^NI
	Slurry	6.63 (1.66–35.00)				
	Environment	1.83 (0.66–5.71)				
**Antimicrobial consumption**	Lincosamides	2.88 (1.45–6.63)	^b^NI	10.98 (3.00–1577.32)	3.79 (2.05–9.80)	^b^NI
	Phenicols	7.57 (1.78–62.24)	^b^NI	^b^NI	6.99 (1.57–47.16)	^b^NI
	Penicillins	2.79 (1.34–6.94)	^b^NI	^b^NI	^b^NI	^b^NI

^a^Antimicrobial: *ERY* Erythromycin, *TET* Tetracycline, *SYN* Quinupristin-dalfopristin, *MDR* Multidrug-resistance, *PNS* Pansusceptibility

^b^NI: not included in the final model

^c^SYN: *E. faecalis* was excluded of the quinupristin-dalfopristin mixed-effects logistic regressions

^d^MDR; ^d^PNS: quinupristin-dalfopristin was excluded for the characterization of MDR and PNS phenotypes in *E. faecalis*

When characterizing the factors associated with the AMR of the 71 *Enterococcus* isolates recovered on intensive farms it revealed that the NWT phenotype was consistently high on farms with high lincosamides consumption for quinupristin-dalfopristin resistance (*p* < 0.01; OR = 5.96; 95% CI = 1.94–423.51), as it was the MDR phenotype (*p* < 0.05; OR = 2.61; 95% CI = 1.27–7.62). An increase in the biosecurity score for disease management was associated with a slightly higher resistance for erythromycin (*p <* 0.05; OR = 1.04; 95% CI = 1.01–1.09) and tetracycline (*p* < 0.05; OR = 1.05; 95% CI = 1.01–1.18), together with a lower PNS (*p* < 0.05; OR = 0.95; 95% CI = 0.85–0.99). No associations were observed for gentamicin.

### Biosecurity scores on intensive swine farms

**Fig. 2:**
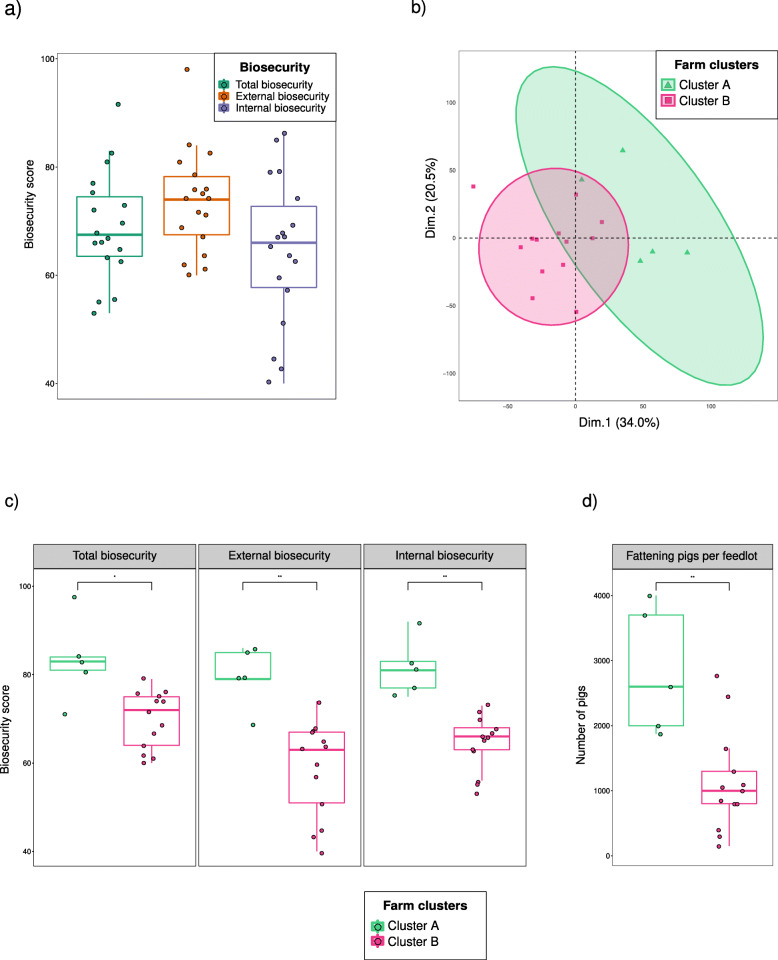
Biosecurity characterization of 18 intensive Spanish pig farms. **a** Boxplots of the total, external and internal biosecurity scores per farm. **b** Principal component analysis followed by an UPGMA hierarchical clustering analysis for grouping farms according to their scores in external and internal biosecurity practices. C) Boxplots of total, internal and external biosecurity scores within each farm cluster. D) Boxplot of the number of fattening pigs per feedlot within each farm cluster. Each farm is represented by a dot with horizontal jitter for visibility. The horizontal box lines represent the first quartile, the median, and the third quartile. Whiskers include the range of points within the 1.5 interquartile range. The differences per cluster were evaluated with the Wilcoxon signed-rank test

The results of the biosecurity questionnaires for 18 intensive farms are presented in Table 4 and Fig. 2a. Within these herds, two types of swine farms were included in the study: 12 finishing and six farrow-to-finish farms. The farm mean biosecurity score was 69.2 ± 10.1. External biosecurity was higher (73.7 ± 9.5) than internal biosecurity (64.6 ± 13.6). The highest internal biosecurity score was achieved in disease management (80.0 ± 21.7), while parameters referring to the measures between compartments, working lines and use of equipment obtained the lowest score (45.7 ± 20.0), with one farm scoring 0 in this subcategory. Among the external biosecurity parameters, the highest score was obtained in the purchase of breeding pigs, piglets and semen (91.7 ± 9.5), being feed, water and equipment supply practices the lowest rated (56.3 ± 17.8).

**Table 4:** Biosecurity scores (Biocheck.UGent™) for the different categories of internal and external biosecurity on 18 intensive Spanish swine farms

		Mean	SD	Median	Min	Max
^a^**Internal biosecurity score**		64.6	13.6	66	40	86
	**Disease management**	80.0	21.7	80.0	20	100
	^c^**Farrowing and suckling period**	64.2	12.1	64.0	50	86
	^c^**Nursery unit**	58.4	11.5	50.0	50	71
	**Fattening unit**	72.6	21.0	79.0	21	100
	**Measures between compartments and the use of equipment**	45.7	20.0	46.5	0	71
	**Cleaning and disinfection**	74.7	21.5	75.0	40	100
^a^**External biosecurity score**		73.7	9.5	74.0	60	98
	**Purchase of animals and semen**	91.7	9.5	96	68	100
	**Transport of animals, removal of manure and dead of animals**	80.2	11.4	79.0	59	100
	**Feed, water and equipment supply**	56.3	17.8	53.0	30	100
	**Personnel and visitors**	63.1	21.7	59.0	29	100
	**Vermin and bird control**	72.8	27.2	80.0	10	100
	**Environment and region**	57.2	28.9	65.0	0	100
^b^**Total biosecurity score**		69.2	10.1	67.5	53	92

^a^Internal and external biosecurity scores are determined by the weighted average of their subcategories. The score for each subcategory is given in a rank from 0 (worst scenario) to 100 (best scenario)

^b^Total biosecurity score is the result of the average of internal and external biosecurity scores

^c^Only six farrow-to-finish intensive farms were included in the study

No significant associations were observed between AMU and the biosecurity scores. When ordinating the farms based on their biosecurity practices, the first two dimensions of the PCA represented the 54.5% of the variability (Fig. 2b). Dimension 1 represented the 34.0% of the variability and it was mainly determined by the vermin and bird control external subcategory (23.7%). Dimension 2 included the 20.5% of the variability and it was predominantly determined by the environment and region external subcategory (63.1%), which refers to the farm location. Based on the farm similarities in biosecurity practices, two clusters of farms were identified: cluster A (5 farms) and cluster B (13 farms) (Fig. 2b). The total, external and internal biosecurity scores in cluster A were significantly higher (*p* < 0.05) than in cluster B (Fig. 2c). The number of fattening pigs per feedlot was significantly higher (*p* < 0.01) on farms with higher biosecurity (cluster A) (Fig. 2d).

## Discussion

A direct transmission of AMR bacteria has been described from pigs and their related farm environment to humans, soil or water [33]. Current policies aim at reducing AMU and we need efficient targets to measure accurately the impact of such interventions. Commensal bacteria, such as *Escherichia coli* or *Enterococcus* spp., are important AMR reservoirs [34] and their use as sentinel microorganisms help to understand time-trends in AMR surveillance [35, 36]. These aspects make these bacteria an excellent tool to evaluate the effect of different on-farm interventions in the AMR threat.

In our study, the main factor that contributed to explain the AMR differences in these bioindicators was the production system. Thus, the chance to find PNS commensal *E. coli* and *Enterococcus* isolates was more than five times higher on organic-extensive than intensive farms. These findings were consistent with the study carried out by Österberg et al. [13], suggesting a lower AMR in commensal *E. coli* recovered from organic pig herds when compared to conventional farming. Since a clear association between AMR and AMU trends in both microorganisms has been previously described [37–39], the most likely explanation for these resistance differences between production systems seems to be the low AMU on organic-extensive farms. Differences in husbandry, such as lower animal density and other risk factors associated with confinement [40], a longer lifespan with less growth pressure, usually slaughter after 14 months of age and a wider behavioral repertoire, which reduces their stress and improves animal welfare, are factors behind this lower AMU.

However, the emergence and spread of AMR bacteria is more complex than a direct antimicrobial selective pressure [13]. In this context, high phenicols use was associated with an increase of six times in ciprofloxacin resistance in commensal *E. coli* recovered from intensive herds, while quinolones use increased the risk of chloramphenicol resistance in these isolates also in nearly six times. This last finding has been previously reported by Murray et al. [41], since ciprofloxacin has been demonstrated to increase relative abundance of chloramphenicol antimicrobial resistance genes (ARGs) in *Enterobacteriacae*. In enterococci, this association between AMR and AMU was revealed mainly by lincosamides use, with an increase in the AMR of nearly two times for erythromycin, 11 times for quinupristin-dalfopristin and four times for the MDR, which was consistent on intensive farms for the last two resistance phenotypes. Cross-resistance among lincosamides, macrolides and streptogramins is common due to *erm* ARGs [42]. In fact, an increased resistance in enterococci to these antimicrobials has been associated with in-feed use of tylosin, a macrolide, and virginiamycin, a streptogramin [43]. Erythromycin resistance in commensal *Enterococcus* was also higher on slurry samples and farms with high penicillins and phenicols consumption. Altogether could be explained by a common oral administration of these compounds [44], which might contribute to the antimicrobial contamination of slurry, and hence, an increase in AMR.

Interestingly, while all *E. coli* were susceptible to last resort antimicrobials forbidden in food-producing animals, such as meropenem or tigecycline, [45], *Enterococcus* resistant to compounds as tigecycline or linezolid were isolated on intensive farms. Tigecycline resistance in enterococci has been associated with certain tetracycline ARGs, as *tet(L)* or *tet(M)*, while linezolid resistance is determined by ARGs that also confer resistance to phenicols, such as the *cfr*-like ARGs, and not only to phenicols, but also to lincosamides, pleuromutilins and streptogramin A, such as the *optrA* ARG [46]. These facts evidenced that cross-resistance to antimicrobials not approved on swine farms can also occur.

Another factor that has been reported as beneficial for AMR control is the application of high standards of biosecurity, mainly by its assistance to reduce AMU to treat bacterial diseases [12]. The biosecurity measures carried out on the intensive farms included in our study were similar to those reported in other European swine herds [5, 23], with a higher internal biosecurity, particularly in the cleaning and disinfection subcategory. Despite high biosecurity has been associated with a reduced AMU [47], no associations were appreciated within our farms, as it was also revealed in a Swedish risk factor study on pig farms [48]. The relationship between biosecurity and AMU seems to be complex. High AMU may lead to an increase in biosecurity standards, while poor biosecurity may be linked to an increased need for antimicrobial treatments. In addition, both AMU and biosecurity are influenced by factors that may act as confounders [49, 50] and thus limit the association between both factors. The only difference observed was that larger herds, determined by the number of fattening pigs per feedlot, had implemented better biosecurity measures when compared with smaller farms, as it has been previously reported [22].

Among the biosecurity measures applied on these farms, a higher cleaning and disinfection score was associated with a slightly lower resistance to sulfamethoxazole and the MDR phenotype in commensal *E. coli*, suggesting that a robust cleaning and disinfection might support AMR mitigation. A correct procedure in these protocols can lead to a reduction in the transmission of bacterial pathogens, as it has been previously described for instance in *Salmonella* spp. removal [51, 52] and, hence, in the AMU. Besides, a higher control of feed, water and equipment supply was associated with a lower gentamicin resistance, as it is recognized that animal feed and water can be a source of AMR bacteria, as *E. coli* or *Salmonella* [53]. In contrast, we observed that a better disease management was associated with a slightly lower PNS and a higher resistance for erythromycin in *Enterococcus* spp., and for tetracycline in both bioindicators. Positive associations between biosecurity and AMR may be linked in some farms to improvements of biosecurity associated to prior health problems and thus to AMU. However, this finding suggests that despite rigorous biosecurity and hygiene measures appear to have important roles in the control and spread of AMR, beyond the prudent and rational AMU, data on AMR control are sparse, and further investigations need to be performed.

## Conclusions

This study proposes that commensal *Escherichia coli* and *Enterococcus* spp. could be considered adequate on-farm bioindicators for the evaluation of factors that contribute to the AMR development. Within these bacteria, AMR was influenced by the production system, with antimicrobial usage as the main differential factor, as it was appreciated for enterococci on farms with high lincosamides usage. Although we did not observe a direct relationship between AMU and biosecurity scores on intensive farms, particular measures, such as adequate cleaning and disinfection protocols, seemed to have an impact in AMR development. However, further investigations need to be implemented for the evaluation of the effect of biosecurity aspects in the AMR threat.

## Data Availability

Not applicable.

## Abbreviations

AIC: Akaike information criterion
AMR: Antimicrobial resistance
AMU: Antimicrobial use
ARG: Antimicrobial resistance gene
BHI: Brain heart infusion
ECOFF: Epidemiological cut-off value
ESVAC: European Surveillance of Veterinary Antimicrobial Consumption
EUCAST: European Committee on Antimicrobial Susceptibility Testing
LRT: Likelihood ratio test
MDR: Multidrug-resistance
MIC: Minimum inhibitory concentration
NWT: Non-wild type
OR: Odds ratio
PCA: Principal component analysis
PNS: Pansusceptibility
UPGMA: Unweighted pair group method with arithmetic mean
WT: Wild type
95% CI: 95% confidence interval
